# Survey of Dental Implant and Restoration Selection by Prosthodontists in Dubai

**DOI:** 10.1155/2021/8815775

**Published:** 2021-08-17

**Authors:** Fatma Al Saleh, Moosa AbuZayeda, Sudarat Kiat-amnuay, Alexander Milosevic

**Affiliations:** ^1^Prosthodontic Unit of the Dental Services Department, Dubai Health Authority, Dubai, UAE; ^2^Department of Prosthodontics, Hamdan Bin Mohammed College of Dental Medicine, Mohammed Bin Rashid University of Medicine and Health Sciences, Dubai, UAE; ^3^Department of General Practice and Dental Public Health, School of Dentistry, University of Texas, Houston, TX, USA

## Abstract

**Background:**

With various surgical and prosthetic component designs being introduced in dental implants, decisions have to be made when choosing a system and a certain prosthodontic protocol. A survey of implant prosthodontic specialists has not been previously performed in the Middle East.

**Aim:**

This study aimed to determine selection criteria and choice of dental implants and restorations by prosthodontic specialists in the Emirate of Dubai, United Arab Emirates.

**Materials and Methods:**

A validated 16-item questionnaire was used in the survey which included demographic information, implant training and experience, implant treatment planning, implant restoration, and implant system preference. The research protocol was approved by the Research and Ethics Committees of Hamdan Bin Mohammed College of Dental Medicine and Dubai Health Authority. Prosthodontists were identified from regulatory authority websites and contacted by e-mail with the questionnaire attached.

**Results:**

A total of 84.6% (77) of the registered prosthodontists in Dubai completed the questionnaire with 66.2% reported practicing implant dentistry. Out of which, 54.9% reported surgically placing dental implants and 45.1% restore them only prosthetically. Prefabricated metal abutments were the most commonly selected abutments for single crowns (76.0%) and for fixed dental prostheses (66.7%). Screw retention is preferred mostly for single crowns (68.0%) and fixed dental prostheses (74.0%). Locators were the most commonly selected type of attachment for implant-retained/supported overdentures (49.0%). Conventional loading was the most selected type of loading in all oral conditions.

**Conclusion:**

Within the limitations of this study, it can be concluded that most prosthodontists in Dubai practice implant dentistry and more than half surgically place dental implants. Prefabricated metal abutments are the most selected type of abutments. Most prosthodontists use screw-retained implant restorations and prefer locator attachments for implant-retained/supported overdentures. Conventional loading is the most preferred implant loading method in all oral conditions. Implant company/system selections are various and there is no major preference for a certain system. The majority of prosthodontists select implant systems based on implant features, literature review, and simplicity of restorative kit.

## 1. Introduction

The introduction of osseointegrated dental implants took place in the early 1980s [1]. It has been reported that between 1 and 2 million dental implants were inserted in the United States in 2010 and the estimated number will increase to 2 to 4 million annually in 2020 [2]. In a retrospective cohort study analyzing the success rates of implant-supported crowns and fixed partial prostheses, it was concluded that implant survival and prosthetic success rates achieved by general dental practitioners in their private practices were lower than those achieved by specialists in university or specialty settings [2]. The prevalence of moderate to severe peri-implantitis in a Swedish population was more likely to occur when the prosthetic treatment is performed by general dental practitioners [3].

Titanium abutments have been considered as the “gold standard” for implant reconstructions due to the high survival rates and favorable mechanical properties [4–6]; however, they have been associated with the grey discoloration of peri-implant tissues [6]. Ceramic abutments have been introduced to provide better aesthetics as they have significantly less mucosal discoloration than metal abutments [7]. In addition, bacterial colonization on zirconia abutments is significantly less than on cast or machined titanium abutments [8]. A systematic review and meta-analysis showed no significant differences between the survival rates and complications of metal and ceramic abutments in single implant-supported prostheses [9]. Prefabricated abutments can only be partially adjusted to meet clinical requirements as ideal placement of the implant fixture is required [10]. Custom implant abutments have been widely used as they allow for the correction of the implant fixture's angle and depth [11] in addition to a better emergence profile [10, 11]. In a retrospective study analyzing loosening of cemented prosthetic reconstructions on prefabricated and custom abutments, it was found that the frequency of prosthetic loosening was greater on prefabricated abutments than on custom abutments and the difference was significant in single crown cases [10].

The type of implant restoration connection (screw or cement-retained) has an effect on multiple clinical and technical aspects of treatment including aesthetics, occlusion, ease of fabrication, retention, retrievability, cost, and even the passivity of the framework [12–14]. According to the systematic review by Wittneben et al., there were no statistically significant differences in the survival and failure rates of screw- and cement-retained implant restorations. Cement-retained restorations had a five-year survival rate of 96.0% and screw-retained restorations had a survival rate of 95.6%; however, cement-retained restorations exhibited more biological and technical complications although ceramic chipping was significantly more frequent in screw-retained restorations [14].

Implant-retained/supported overdentures require careful evaluation and thorough treatment planning to achieve optimal clinical results [15]. Other than determining the number of implants to restore an edentulous patient, a decision has to be made on the type of attachment to provide retention of the prosthesis to the implants. Rigid attachments restrict rotation such as telescopic copings and U-shaped bars while resilient attachments allow various degrees of rotation and some degree of angulation correction such as round-shaped bars and clips, balls, magnets, and locators [16]. In maxillary implant-supported overdentures, locators have demonstrated superior clinical results over 3 years in terms of better peri-implant hygiene, ease of denture preparation, reduced frequency of maintenance, and cost [17]. A prospective 5-year clinical study which compared ball and telescopic attachments in mandibular implant-supported overdentures found that ball attachments were associated with greater maintenance needs specifically in the first 1 to 3 years of follow-up [18].

Modifications to the surgical and loading protocols have evolved since the early 1990s [19]. The 4th ITI Consensus Conference classified loading protocols as conventional, early, and immediate [20]. The predictability of loading protocols can be influenced by multiple factors such as occlusion, periodontal health, parafunctional habits, implant site features, implant size, and properties, in addition to the timing and method of implant placement [20]. In a systematic review comparing implant success rates with different loading protocols, no statistically significant differences in implant success rates were reported [21]. A multicenter randomized controlled trial found all loading strategies to be successful 4 months postloading and it was concluded that the most relevant factor in achieving such results was ensuring high insertion torque during implant placement (40 Ncm or higher) [22].

Cardoso et al. in 2013 surveyed members of the American College of Prosthodontists (ACP) and American Academy of Maxillofacial Prosthetics (AAMP) to identify the most commonly used implants and the overall restorative preference and found that prosthodontists' selections were based on training, implant features, and literature support [23]. They also found that 79% of respondents were trained to use Nobel Biocare and this implant was the most commonly used system in all clinical situations. Another study surveying prosthodontists in the United States of America (US) regarding their implant experience showed that most prosthodontists (82%) used implant-supported restorations in their practice. However, most implants restored by prosthodontists were placed by nonprosthodontists [24]. In 2010, it was reported that the three most widely used implant systems among US dental schools were Nobel Biocare, followed by Biomet 3i and Straumann, and the most commonly used luting agent for implant restorations' cementation was resin-modified glass ionomer cement [25]. In 2016, it was reported that the most commonly used implant systems in New Zealand were Nobel Biocare, Biomet 3i, Straumann, and Neoss while the main factors for selection were reliability, ease of use, familiarity, and predictability [26].

A review of the literature did not find a published survey on dental implants and restorations selected by prosthodontic specialists in the Middle East. The only published study, in 2013, compared dental implant use among private dental practitioners in the United Arab Emirates (UAE) to those in Iran where ITI was reported as the most widely used implant system in the UAE and Implantium in Iran [27]. Therefore, the aim of this study was to determine selection criteria and choice of dental implants and restorative preference by prosthodontic specialists practicing in the Emirate of Dubai, UAE. The trends of practicing implant dentistry, the types of implants/implant restorations, the criteria of selection, the types of loading protocols, and the use of implant planning software among prosthodontists in Dubai were explored.

## 2. Materials and Methods

The 16-item questionnaire used in this study was based on the one developed by Cardoso et al. [23]. Although the questionnaire itself was not published, the corresponding author was contacted and a copy was obtained. With the approval of the prosthodontic faculty at the Hamdan Bin Mohammed College of Dental Medicine (HBMCDM), the 22-item questionnaire used in Cardoso's study was modified slightly in order to make it shorter. The questionnaire included demographic information, implant training and experience, implant treatment planning, implant restorations, implant system preference, and selection, in addition to implant loading protocol (see the supplementary file). The research study was approved by the Research and Ethics Committee of HBMCDM in addition to Dubai Scientific Research Ethics Committee of Dubai Health Authority (DHA) in December, 2016 (DSREC-SR-12/2016_03). The lists of registered prosthodontists practicing in Dubai were accessed through the official websites of the licensing bodies: DHA and Dubai Health Care City (DHCC). The information obtained from the lists included prosthodontists' names, clinic names, contact numbers, and addresses. In January, 2017, an e-mail with an attached questionnaire requesting the recipient's participation in the study was sent. A second e-mail was sent one month later to those who did not respond to the initial e-mail. In order to encourage participation, prosthodontists were contacted via phone and some were visited in their clinical practices. Responses to the questionnaires were gathered by the 30th of April, 2017. The target sample of the study included all prosthodontic specialists working in DHA, DHCC, and private clinics located in Dubai. The study excluded prosthodontic specialists who did not wish to participate in the study, general dental practitioners, and nonprosthodontic specialists placing and/or restoring implants in Dubai. The aim was to match the target sample to that in Cardoso's study (prosthodontists only).

### 2.1. Demographic Information

The demographic information gathered was the year of graduation from dental school and whether or not the prosthodontist practiced implant dentistry. Prosthodontists who did not practice implant dentistry were informed not to continue completion and return the questionnaire. Prosthodontists who surgically placed implants were identified.

### 2.2. Implant Training and Experience

Information regarding implant training including the types and duration of implant training programs was gathered. The duration of practicing implant dentistry was also assessed.

### 2.3. Implant Treatment Planning

Prosthodontists' participation in implant treatment planning with other specialties was surveyed in addition to the frequency of using implant planning software programs.

### 2.4. Implant Restorations

The types of abutment used for single implant-supported crowns and implant-supported fixed dental prostheses were assessed. The choices given were prefabricated metal abutments, prefabricated ceramic abutments, cast to gold/UCLA abutments, and CAD/CAM (custom) abutments. The types of attachment used for implant-supported/retained overdentures were also surveyed and included bar/clip attachments, ball/socket attachments, locators, and telescopic and magnetic attachments. Finally, the type of implant superstructure retention (screw or cement retention) was also assessed. Respondents were asked to select one answer per question.

### 2.5. Implant System Preference and Selection

The implant systems most often used in different oral conditions were assessed including anterior areas (incisors and canines), posterior areas (premolars and molars), edentulous arches, and the overall preferred choice of implant systems. The major implant companies included were Astra Tech, Ankylos, Xive, Bio Horizon, Neoss, Biomet 3i, Nobel Biocare, Straumann/ITI, and Zimmer. One more option given was “other” and the implant company had to be specified. Respondents were asked to select one implant system for every condition. In addition, respondents were asked to rank the criteria when selecting an implant company/system in order of importance. Nine criteria were included: general implant features, simplicity of surgical kit, simplicity of restorative kit, literature support, proven aesthetic outcome, customer service/product support, cost, education support from the implant company (provider), and educational background (system used during training).

### 2.6. Implant Loading

The preferred loading protocols were surveyed in different oral conditions: anterior (incisors and canines), posterior (premolars and molars), and edentulous arches. One loading protocol (immediate/early/conventional) was to be selected for every condition. Immediate loading was defined as loading the implants within 1 week postplacement. Early loading was defined as loading the implants 1 week and before 2 months postplacement. Conventional loading was defined as loading the implants 2 months postplacement. The respondent was asked to select the main reason for not loading implants immediately and the options included patient factors (smokers, uncontrolled diabetics, bruxists, etc.), lack of education/training on immediate loading, when additional surgeries (such as bone augmentation or sinus lifting) are performed, disagreement with immediate loading concept, or other reasons. Respondents were asked to select one main reason and to write in the reason if the option was not present.

Data were entered in the computer using SPSS for Windows version 20.0 (SPSS Inc., Chicago, IL). Differences in frequency of categorical variables were tested using chi-square with significance set at *P* < 0.05.

## 3. Results

Out of the 91 prosthodontists registered in Dubai, 8.8% (8) refused to participate and 6.6% (6) failed to respond. Thus, a total of 84.6% (77) prosthodontists responded to the questionnaire. Of these, 33.8% (26) reported not practicing implant dentistry and 66.2% (51) reported they did. Unanswered questions were considered as missing values and were excluded from the results.

### 3.1. Demographic Information

Table 1 shows the demographic information of the respondents. Out of the prosthodontists practicing implant dentistry, 66.7% (34) were working in private clinics, 21.6% (11) worked in DHA, and 11.7% (6) worked in DHCC. In addition, 54.9% (28) of those practicing implant dentistry reported surgically placing and restoring implants while 45.1% (23) reported restoring them only prosthetically. The difference in surgical implant placement between prosthodontists who graduated in the 2000s (56.9%) compared to those who graduated in the 1970s–1990s (43.1%) was not statistically significant (*P*=0.964).

### 3.2. Implant Training and Experience

Among the different types of implant training programs, 52.9% (27) of the prosthodontists selected a combination of prosthodontic residency and implant fellowship or continuing education courses, 35.3% (18) selected prosthodontic residency training, 9.8% (5) selected continuing dental educational courses, and only 2.0% (1) selected other training programs. The duration of training varied between the respondents where 13.0% (6) of the prosthodontists trained for 2 years or less, 80.3% (37) trained for more than 2 years and up to 4 years, and 6.5% (3) trained for more than 4 years. The mean duration for practicing implant dentistry among the participants was 10.7 years (SD 6.1).

### 3.3. Implant Treatment Planning

All of the prosthodontists (100%) reported taking part in patients' implant treatment planning when working with other specialties. Fifty-three percent of the prosthodontists (27) reported “limited/no use” of implant planning software while 43.1% (22) reported using them in “special cases” and only 3.9% (2) reported using them “always.”

### 3.4. Implant Restoration

**Figure 1:**
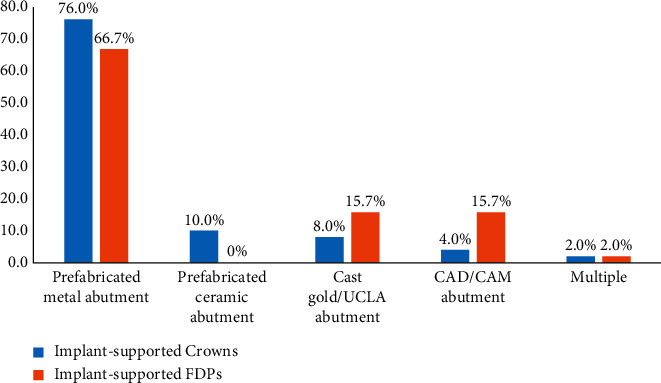
The types of crowns and fixed dental prostheses (FDPs) abutments.

**Figure 2:**
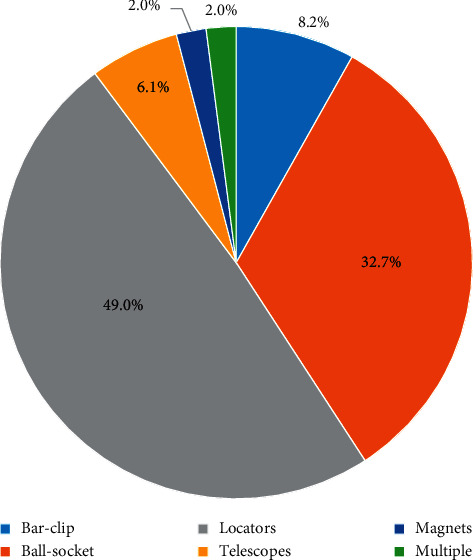
The types of attachments for implant-retained/supported overdenture.

The most commonly selected abutment type for single implant-supported crowns and fixed dental prostheses (FDPs) was the prefabricated metal abutment, 76% (38) and 67% (34), respectively. The prefabricated ceramic abutment was selected only for implant-supported crowns and none of the prosthodontists reported using it for FDPs. Both cast gold/UCLA and CAD/CAM abutments were selected for implant-supported FPDs more than for crowns. There were only 2% (1) of respondents who selected more than one abutment type. The detailed results are shown in Figure 1. Sixty-eight percent (34) of the prosthodontists reported using screw-retained crowns while 32.0% (16) reported using cement-retained crowns. Similarly, in FDPs, 74.0% (37) reported using screw retention rather than cement retention which were selected by 26.0% (13). For implant-retained/supported overdenture attachments, locators were the most commonly selected type of attachment, followed by ball-socket attachments. Both attachments were reported to be used by more than 80% (40) of respondents. There were only 2% (1) of respondents who selected more than one attachment. The detailed results are shown in Figure 2.

### 3.5. Implant System Preference and Selection

Table 2 presents the complete results of the used implant systems in different oral regions. The criteria of selecting implant systems by prosthodontists are displayed from most important to least important in Table 3.

### 3.6. Implant Loading

Conventional loading was the most selected type of loading protocol used in all oral conditions: 52.9% (27) in anterior areas, 86.3% (44) in posterior areas, and 76.5% (39) in edentulous arches.

**Figure 3:**
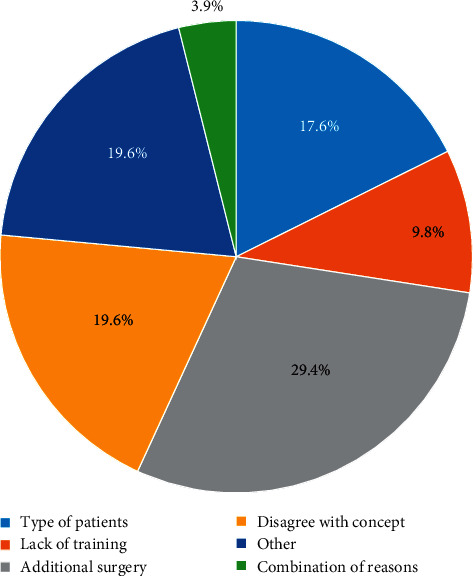
The reasons reported by prosthodontists when immediate loading is not used.

In anterior areas, immediate loading was reported by 39.2% (20) while 7.8% (4) reported using early loading. In posterior areas, 5.9% (3) of the prosthodontists reported using immediate loading while 7.8% (4) reported using early loading. In edentulous arches, 17.6% (9) of the prosthodontists reported using immediate loading while 5.9% (3) reported using early loading. The most common reasons behind not using immediate loading are shown in Figure 3.

## 4. Discussion

The aim of this study was to determine selection criteria and choice of dental implants and restorations by prosthodontists in Dubai. The response rate of 84.6% is higher than previous similar studies (18% to 71%) [23–26] perhaps due to the smaller sample size in Dubai.

Among the respondents, 33.8% reported not practicing implant dentistry while this was reported to be 17.8% among prosthodontists practicing in the US [24]. This may be due to the lack of implant training during prosthodontic postgraduate studies or the delivery of implant treatment by other specialists such as oral surgeons, periodontists, or general dental practitioners who have undergone implant training. In contrast, 54.9% reported surgically placing implants which is higher than the rates reported in the US by Eckert et al. [24] in 2002 (12%) and by Cardoso et al. in 2013 (39%) [23].

The majority of respondents (92.2%) reported graduating in the 1990s–2000s while only 7.8% reported graduating in the 1970s–1980s, therefore the influence of the years of graduation on the trends of implant/restoration preferences could not be assessed.

The most frequently selected implant training program was a combination of prosthodontic residency and implant fellowship or continuing education courses. That the “educational background/system used during training” criteria did not have a major role in selecting an implant system was unexpected. For the majority of respondents (80.3%), the reported implant training duration ranged between 2 and 4 years which is similar to the results reported by Cardoso et al. in the US [23].

Dental implant treatment has become more predictable due to the advances in digital technology [28]. However, only 3.9% of the respondents reported using implant planning software “always” and 52.9% reported “limited/no use,” while 43.1% reported use “in special cases.” The rates of using such software among prosthodontists in the US were higher, with 11% of the respondents reported using them “always,” 54% using them “sometimes,” and 35% “never” using them [23].

Due to its favorable mechanical properties, titanium abutments have been considered as the “gold standard” abutments for implant reconstruction [4–6]. In this study, the prefabricated metal abutment was the most commonly selected type of abutment for single implant-supported crowns (76.0%) and for fixed dental prostheses (66.7%). In the study by Cardoso et al., abutment style preferences were presented according to oral regions. Custom-milled ceramic abutments were mostly preferred in anterior and highly aesthetic areas and prefabricated metal abutments were preferred in posterior areas [23]. Probable reasons behind the preference for prefabricated abutments over custom abutments (cast or CAD/CAM fabricated) in this study are shorter fabrication time and reduced cost.

Screw-retained restorations offer the benefits of retrievability of the super structures for repair, hygiene, and abutment screw tightening in addition to placement in limited interarch spaces [12, 13]; however, they require accurate implant placement and the screw access hole can compromise aesthetics and occlusion [12]. Most of the respondents in this study reported using screw retention for implant-supported crowns (68.0%) and implant-supported fixed dental prostheses (74.0%). For implant-retained/supported overdentures, unsplinted attachments offer improved access for oral hygiene measures, reduced cost, and ease of denture preparation while splinted attachments allow for better force distribution and can compensate for misaligned implants [16]. The majority of respondents (89.9%) reported using unsplinted attachments most often. Locators were the most commonly selected attachment type (49.0%) followed by the ball-socket type (32.7%). Similar results were reported by Cardoso with 77% of respondents preferring stud (unsplinted) attachments and 86% preferring locators [23]. It seems that locators are the most preferred attachment type due to their simplicity and ease of maintenance as well as the availability of different inserts with different ranges of retention and angulations [15, 16].

With the presence of various implant systems in the market, selection among all the respondents varied and there was no major preference for a certain company/system. Ankylos was the most popular implant system (25.5%) in anterior regions while Xive was the most commonly selected in posterior regions (19.6%) and in edentulous arches (23.5%). As an overall preference, 17.6% selected Nobel Biocare. Different results were reported in an earlier study in the UAE where the most commonly preferred systems were ITI followed by Implantium and SPI [27].

The various implant systems selected by the respondents in this study clearly demonstrate a lack of preference for a certain company/system. The three most important criteria behind selecting implant systems were general implant features followed by literature support and simplicity of restorative kit. Training background, educational support from the provider, and cost were selected as the least important criteria. Such results are similar to those reported by Cardoso et al. [23], where the top three criteria for selecting implant systems among the respondents in the US were implant features, literature support, and simplicity of restorative kit. Cost and simplicity of the surgical kit were reported as the least important criteria. Another study in New Zealand [26] reported reliability, ease of use, and familiarity as the main factors behind system selection and cost as the least important which is similar to this study.

Multiple studies have discussed the predictability of immediate and early loading methods in comparison to the conventional method of loading dental implants [20, 22, 29, 30]. No significant differences in the clinical outcomes between the loading protocols were reported [22, 29]. In this study, conventional loading was the most common type of loading in all oral conditions: 52.9% in anterior areas, 86.3% in posterior areas, and 76.5% in edentulous arches. Immediate loading was the second preferred method in anterior areas (39.2%) and in edentulous arches (17.6%). The most common reason behind not using immediate loading was “when additional surgery is performed,” reported by 29.4%. Cardoso et al. also reported conventional loading as the most common type of loading and 55% of respondents who do not practice immediate loading reported that reason behind that is not believing in the immediate loading philosophy [23].

Although the response rate for this survey study is considered very high (84.6%), there is a limited number of prosthodontic specialists in the Emirate of Dubai with only two-thirds practicing implant dentistry. Future studies with a similar survey would be beneficial to compare the trends of implant and restoration selection by prosthodontists in different regions of the world.

## 5. Conclusions

Within the limitations of this study, the following conclusions can be drawn:

- Most prosthodontists in Dubai practice implant dentistry and more than half surgically place dental implants.
- Prefabricated metal abutments are the most selected type of abutments and most prosthodontists use screw-retained implant restorations.
- Locators are the most commonly selected type of attachments for implant-retained/supported overdentures.
- Conventional loading is the most preferred implant loading method in all oral conditions.
- Implant company/system selections are various and there is no major preference for a certain system. The majority of prosthodontists select implant systems based on implant features, literature review, and simplicity of restorative kit.

## Figures and Tables

**Table 1:** Demographic details of respondents.

Item	No. (%)
*Gender*	
Male	44 (57.1%)
Female	33 (42.9%)

*Graduation years*	
1970s	2 (2.60%)
1980s	4 (5.20%)
1990s	31 (40.2%)
2000s	40 (52.0%)

*Implant dentistry practice*	
Prosthodontists not practicing implant dentistry	26 (33.8%)
Prosthodontists practicing implant dentistry	51 (66.2%)
(i) Surgical and prosthetic implant practice	28 (54.9%)
(ii) Prosthetic implant practice only	23 (45.1%)

**Table 2:** Prosthodontists' preferred implant systems.

Implant systems	Anterior regions (%)	Posterior regions (%)	Edentulous arches (%)	Overall preference (%)
Ankylos	25.5	11.8	5.9	7.8
Xive	5.9	19.6	23.5	15.7
Bio Horizon	5.9	5.9	2.0	3.9
Neoss	0	3.9	0	2.0
Biomet 3i	3.9	2.0	5.9	2.0
Nobel Biocare	15.7	11.8	17.6	17.6
Straumann/ITI	13.7	11.8	9.8	15.7
Zimmer	9.8	11.8	11.8	13.7
Southern Implants	5.9	5.9	5.9	5.9
Osstem	3.9	3.9	3.9	3.9
Microdent	2.0	2.0	2.0	2.0
SGS	2.0	0	2.0	2.0
SPI	2.0	2.0	2.0	2.0
Myriad	2.0	2.0	0	0
Slock	2.0	2.0	2.0	2.0
Bicon	0	3.9	5.9	3.9
Dentium	0	0	2.0	0

**Table 3:** The ranking of criteria when selecting an implant company/system.

Criteria of selecting an implant system (from most important to least important)
(1) General implant features (surfaces, body, and abutments)
(2) Literature support
(3) Simplicity of restorative kit
(4) Proven aesthetic outcome
(5) Customer service/product support
(6) Simplicity of surgical kit
(7) Cost
(8) Educational support from provider (company)
(9) Educational background (system used during training)

## Data Availability

The datasets used to support the findings of this study are available from the corresponding author upon reasonable request.
