# Supplementary material for: Survey of Dental Implant and Restoration Selection by Prosthodontists in Dubai
Source: Int J Dent. 2021 Aug 17;2021:8815775. doi: 10.1155/2021/8815775 (PMC8387181; doi:10.1155/2021/8815775)
Supplement: Supplementary Materials — The 16-item questionnaire used in this study is provided. [file 8815775.f1.pdf]

SURVEY QUESTIONS

1. Which year did you graduate from dental school? _______________

2. Do you place implants? ☐ Yes ☐ No

3. Do you restore implants? ☐ Yes ☐ No

If ***no*** to question 3, please **stop** and **thank you** for taking the time to do the survey. Kindly, **send** back the questionnaire with your answers above.

If ***yes*** to question 3, please continue to question 4.

4. How many years have you been practicing implant dentistry? _________________

5. Which type of implant training program did you have? (Select all that apply) and please specify the duration of the program in years or months or days.
   - ☐ Prosthodontic Residency: __________________
   - ☐ Implant Fellowship: __________________
   - ☐ Continuing Dental Education Courses
   - ☐ Other (please specify) ___________________________________

6. If you work with other specialties, do you take part in the patient's implant treatment planning before implant surgery?
   ☐Yes ☐ No

7. How often do you use implant planning software (i.e. Facilitate, Simplant ...etc)?
   - ☐ Always
   - ☐ Use only for special cases
   - ☐ Limited use/ do not use at all

8. What type of **abutments** do you use most often to fabricate **single implant-supported crowns**?
   - ☐ Pre-fabricated metal abutments (Ti, gold)
   - ☐ Pre-fabricated ceramic abutments (Zirconia, Alumina)
   - ☐ Angulated abutments
   - ☐ Cast-to gold/UCLA abutments
   - ☐ CAD/CAM abutments

9. What type of **abutments** do you use most often to fabricate **implant-supported fixed dental prosthesis**?
   - ☐ Pre-fabricated metal abutments (Ti, gold)
   - ☐ Pre-fabricated ceramic abutments (Zirconia, Alumina)
   - ☐ Angulated abutments
   - ☐ Cast-to gold/UCLA abutments
   - ☐ CAD/CAM abutments

10. What type of **attachments** do you use most often when planning an **implant supported/retained denture**?
   - ☐ Bar and clip attachment
   - ☐ Ball and socket attachment
   - ☐ Locator attachment
   - ☐ Telescopic attachment
   - ☐ Magnetic attachment

11. What type of fixed implant restorations do you use most often in **single implant cases**?
   - ☐ Screw-retained
   - ☐ Cement-retained

12. What type of fixed implant restorations do you use most often in implant-supported **fixed dental prosthesis cases?**
   - ☐ Screw-retained
   - ☐ Cement-retained

13. Which loading protocol do you prefer in the following conditions? Please mark with an **X.** Mark **<u>one only per row</u>**.

	**Immediate loading (earlier than 1 week)**	**Early implant loading (1 week to 2 months)**	**Conventional loading (more than 2 months)**
Anterior (incisors and canines)			
Posterior (premolars and molars)			
Edentulous patients			

14. If immediate loading is not used, which of the following is the main reason?
   - ☐ Type of patient that presents to practice (smokers, uncontrolled diabetics, bruxists..)
   - ☐ Lack of education/training
   - ☐ Administration does not allow
   - ☐ When additional surgeries (bone augmentation, sinus lifting... etc.) are performed
   - ☐ Disagree with immediate loading concept
   - ☐ Other (please specify) ____

15. Which implant system do you use most often in the following situations? Please mark with an **X.** Mark **one only per row**.

	**Astra Tech**	**Ankylos**	**Xive**	**Bio Horizon**	**Neoss**	**Biomet 3i**	**Nobel Biocare**	**Mini Implant System**	**Strauman ITI**	**Zimmer**	**Other Please specify**
Anterior (incisors & canines)											
Posterior (premolars & molars)											
Edentulous patients											
Overall preference (1st choice)											

16. Next to the following criteria, please **rank** them in order of importance **(from most important=1 and least important=9**) when selecting an implant company/system.

General implant features (surfaces, body, abutments)	
Simplicity of surgical kit	
Simplicity of restorative kit	
Literature support	
Proven esthetic outcome	
Customer service/Product support	
Cost	
Educational Support from provider (company)	
Educational background (system used during training)	

Thank you
